# Targeted Therapeutic Approach Employing *In Silico* Methods to Identify Potent Flavonoids in Cervical Cancer

**DOI:** 10.2174/0118715303362788250616052258

**Published:** 2025-06-30

**Authors:** Srishti Sharma, Anuja Mishra, Pratibha Pandey

**Affiliations:** 1 Department of Biotechnology, GLA University, Mathura, India;; 2 University Centre of Research and Development, University Institute of Biotechnology, Chandigarh University Gharuan, Mohali, Punjab, India;; 3 Centre for Research Impact and Outcome, Chitkara University Institute of Engineering and Technology, Chitkara University, Rajpura- 140401, Punjab, India;; 4 Center for Global Health Research Saveetha Medical College, Saveetha Institute of Medical and Technical Sciences, Chennai, India

**Keywords:** Flavonoids, naringenin, notch, cell signaling pathway, *in silico*, cervical cancer

## Abstract

**Background:**

The prevalence of cervical cancer has substantially increased, leading to global apprehension. The signaling pathway governs many cellular differentiation processes that occur during embryonic development and adulthood.

**Aims:**

Our study has mostly focused on discovering inhibitory plant chemicals, specifically flavonoids, that can block the Notch signaling pathway owing to the numerous adverse effects associated with standard treatments. Thus far, only a few studies have documented these specific flavonoids' ability to inhibit the three essential targets of the Notch signaling system in cervical cancer.

**Methods:**

This study aimed to assess the inhibitory potential of twenty-five potent flavonoids against the Notch signaling pathway components in cervical cancer using various *in silico* methods.

**Results:**

Of all the compounds tested, naringenin exhibited the most favorable binding energy (with concrete free binding energy value) against jagged2, notch 2, and notch 3 in cervical cancer.

**Discussion:**

Naringenin has been reported with significant anticancer potential in several carcinomas and hence we have selected for its evaluation against cervical cancer. Amongst all, naringenin has shown significant anticancer potential. However, significant amount of experiments including clinical studies are needed to validate its candidature for cervical cancer.

**Conclusion:**

Naringenin could further be evaluated for its anticancer potential in cervical cancer *via* employing *in vitro* approaches. To overcome the limitations of current chemotherapeutic procedures, developing naringenin as a novel anticancer drug requires a systematic and strategic approach to rational drug discovery that is both efficient and cost-effective.

## 1 INTRODUCTION

The Notch signaling system is crucial for controlling several cellular activities, including proliferation, survival, differentiation, and death [1]. The significance of this pathway in drug-resistant tumor cells has been emphasized in newly published studies. Evidence has indicated that blocking this pathway can enhance drug sensitivity and effectively reduce cancer cell proliferation, invasion, and metastasis. Several studies have examined the correlation between increased Notch expression and the occurrence of cervical cancer. These findings motivated us to investigate the potential of targeting the Notch signaling pathway as a treatment for cervical cancer [2]. Owing to continuous shifts in population and illness trends, the global effect of cancer is rapidly increasing. Cervical cancer rates vary significantly between economically developed and underprivileged countries, reflecting the overall worldwide impact of cancer. Most human cancers are thought to be initiated by viral infections, which may accelerate the progression of cancer in different phases. There are approximately 15 variants of Human Papillomavirus (HPV) that are linked to the formation of cancer [3]. Despite the efficacy of screening programs, cervical cancer is a prominent public health concern. However, finding cures for cancer is a lengthy and demanding procedure that requires significant financial resources. Consequently, there is growing worldwide interest in tackling the constraints associated with traditional chemotherapeutic approaches. The utilization of *in silico* technology and the extraction of medicinal properties from plant-derived chemicals have emerged as crucial methods in the field of drug development. These are some of the recent computational methods applied to the prediction of interactions of molecules inside biological systems; thus, much has been considered about molecular recognition and the better design of a more potent bioactive compound. In fact, a modern computational approach known as molecular docking explains receptor protein-ligand interactions. It gives knowledge on the stabilization of complexes. These methods target protein-ligand complexes as a basis to study the mechanism of binding between ligands and protein receptors for the discovery and development of new bioactive compounds to fight against diseases. To better accurately forecast the binding interactions between ligands and target proteins, we employ flexible molecular docking in our research. Making sure the protein and ligand structures are precisely tuned and energy-minimized is the first step in the procedure. By evaluating various interactions between the ligand and the flexible protein binding site, including hydrogen bonding, hydrophobic forces, van der Waals interactions, and electrostatic forces, the docking algorithm then investigates other possible binding poses. Flexible docking provides a more accurate depiction of the binding process by integrating these interactions in a dynamic setting. In this study, the main advantage of flexible docking is its improved capacity to forecast modes, which accurately captures the fluid character of protein-ligand interactions [4].

Numerous studies have offered data to support the theory that phytochemicals can stop cervical cancer progression by altering different signaling pathways [5]. Phytochemicals derived from plants have garnered increasing attention in the quest for effective therapeutic strategies against various carcinomas, offering significant health benefits. Toxicity in several chemotherapy regimens resulted in the desire to find better anticancer drugs. Studies show that natural phytocompounds are effective substitutes for chemotherapeutic drugs and surgical procedures [6].

Plant-derived compounds have the potential to combat cancer by inhibiting the growth of malignant cells or altering several signaling pathways. Flavonoids have garnered significant interest from researchers because of their multitude of biological benefits, including anti-inflammatory, anti-allergic, antioxidant, anti-angiogenic, anti-mutagenic, antibacterial, and anticancer properties [7]. These molecules are derived from plants and have distinct biological advantages. The main subcategories of flavonoids include flavonols, flavanones, isoflavones, chalcones, flavanonols, and flavonols. Their ability to induce apoptosis, interfere with the cell cycle, and inhibitition of angiogenesis renders them valuable instruments for cancer management [8].

Therefore, it is essential to identify powerful molecular targets that can impede the progression of cervical carcinogenesis. Furthermore, it is crucial to include plant-derived or dietary drugs in chemoprevention efforts. Recent advancements in cervical cancer therapy strategies have led to the consideration of phytochemicals as viable means to inhibit the Notch signaling pathway, which is implicated in various forms of carcinomas [9]. Currently, only a limited number of natural medicines targeting the Notch signaling system in cervical cancer have been identified. Therefore, using a computer-based method, our study aimed to clarify the mechanism by which flavonoids that target the Notch signaling system are involved in cervical cancer [10].

## 2 MATERIALS AND METHODS

### 2.1 Preparation of Ligands (Flavonoids)

The structures (.sd) of the identified compounds were sketched using ChemDraw and used as ligands (Table ****1****). The SD files were converted to their corresponding Three-Dimensional (3D) structures and saved in .pdb format using Open Babel. Gasteiger charges were added, and non-polar hydrogens were merged using AutoDock4.2 [11].

### 2.2 Production of Target Protein (Macromolecule)

The 3D crystal structures of jagged2 (PDB ID: 5MW7) with resolution of 2.80 Å, notch 2 (PDB ID: 5MWB) with a resolution of 1.86 Å, and notch 3 (PDB ID: 4ZLP) with resolution of 2.479 Å were acquired from the Protein Data Bank (PDB) website at http://www.rcsb.org/pdbin.pdbformat [12]. All the 3D protein structures were subjected to refinement and energy minimization prior to conducting the docking analyses. The whole PDB structures of the proteins were used in performing the molecular docking studies. These involved the following: adding the missing atoms of residues, polar hydrogen atoms, and Kollman charges and removal of crystallographic water molecules, extraneous ligands, and irrelevant ions present in the structures of the proteins. The protein structures were cleaned, water molecules removed, Gasteiger charges computed, polar hydrogens added, and non-polar hydrogens merged using AutoDock4.2. The ligand with the most negative binding energy was found to have the highest docking score to the target protein of interest [13].

### 2.3 Active Site Prediction

The possible active binding sites of the proteins were obtained using DoGSiteScorer. The binding sites with the best volume, surface area, and druggability score were selected for this study. DoGSiteScorer is a grid-based method, which uses a difference of Gaussian filter to detect potential binding pockets, solely based on the 3D structure of the protein, and splits them into subpockets [14].

### 2.4 Molecular Docking Analysis

AutoDock 4.2 was utilized to convert both receptor and ligand structures into the pdbqt file format, incorporating atomic charges, atom-type definitions, and, for ligands, topological information, including rotatable bonds. A docking grid was centered to ensure comprehensive coverage of the receptor's binding site. Docking simulations were carried out using AutoDockVina, which generated multiple ligand conformations within the receptor complex. These confirmations were subsequently ranked based on their binding energy to determine the most favorable interactions. Additionally, docking of target proteins and ligands was performed using iGEMDOCK (version 2.1). The Genetic Algorithm (GA) parameters, which guided the docking procedure, were set as 200 (population size), 70 (generations), and 3 (number of solutions). Various binding interactions, including Hydrogen bonding (Hb), van der Waals forces (vdW), and electrostatic interactions, were analyzed to characterize ligand-receptor binding. Finally, the docked conformations were visualized using Discovery Studio Visualizer and PyMol to examine binding orientations and molecular interactions in detail [15]. The results in Tables **2**, **3**, **4**, and **5** indicate the docking score of isolated compounds and standard drugs as simulated by AutoDockVina.

### 2.5 Conduct PASS (Prediction of Activity Spectra for Compounds) Analysis

The PASS analysis was conducted to forecast the biological activity range of a phytocompound, taking into account its SAR (Structure-Activity Relationship) with a recognized chemical entity. A combination of online and offline technologies was utilized to compute the drug-likeness of the chosen phytocompound. The drug-likeness of the screened compounds was determined using Swiss ADME [16]. The information is presented in Table **6**. The ligands underwent Lipinski, Ghose, Veber, Egan, and Muegge screening. The parameters taken into account for evaluating drug-likeness were Molecular Weight (MW), the count of hydrogen bond donors, the logarithm of the partition coefficient (log P), the number of hydrogen bond acceptor sites, the count of rotatable bonds, and the Topological Polar Surface Area (TPSA) [17].

### 2.6 Elucidation of Pharmacokinetic Parameters Analysis

The ADME (Absorption, Distribution, Metabolism, and Excretion) properties of all the components, including both standard medicines and chosen phytocompounds, were predicted using the SwissADME software (http://www.swiss adme.ch/). The information is presented in Table **7**. This web-based application evaluates various essential pharmacokinetic characteristics of a substance, including its capacity to cross the Blood-Brain Barrier (BBB), its permeability through the skin (LogKp), and its impact on the inhibition of Cytochrome P450 enzymes and its interaction with P-glycoprotein (P-gp) as a substrate [18].

## 3 RESULT

### 3.1 Conducting Docking Study of Certain Flavonoids Against Notch Targets

Molecular docking is a crucial *in silico* technique used to predict the mode of interaction between a small ligand and its target protein within an established binding site. One of the key parameters in molecular docking studies is the binding energy, which provides insight into the strength and affinity of a compound’s interaction with the target protein. A lower binding energy signifies a stronger interaction, making the compound a more favorable drug candidate, whereas a higher binding energy indicates a weaker binding potential. Docking score is assessed to determine the strength of the molecular interactions or bonding between the ligand and the protein during the formation of the complex structure. Different docking programs utilize distinct methodologies to evaluate these interactions. For instance, AutoDockVina employs a sophisticated gradient optimization method during its local optimization procedure to enhance the accuracy of docking score predictions. In contrast, iGEMDOCK utilizes an empirical scoring function alongside an evolutionary approach to evaluate ligand-protein interactions [19]. Notably, these compounds exhibited stronger docking scores than cisplatin, a well-known drug inhibitor of cervical cancer, which demonstrated a relatively weak docking score of -1.2 kcal/mol. Several amino acid residues within the protein's binding pocket interact with the ligand, varying depending on the specific phytochemical bound. One such residue exhibits amphipathic properties, featuring a long hydrophobic carbon tail near the backbone and a positively charged amino group on its side chain. It is commonly found in protein binding sites, where it forms hydrogen bonds with ligands and plays a role in acid-base catalysis, making it a promising drug target. The interaction stability between the ligands and the active site of the target proteins was facilitated by various non-covalent interactions, including hydrogen bonding, van der Waals forces, and different types of π interactions. These stabilizing forces contributed significantly to the proper orientation and docking of the ligands within the protein’s binding pocket. The presence of these non-covalent interactions between the ligands and specific amino acid residues within the active site further reinforced the binding stability, thereby enhancing the potential therapeutic efficacy of the investigated compounds.

### 3.2 PASS Analysis Result of Selected Phytocompound

Lipinski's rule of five elucidates the essential molecular characteristics of a chemical that play a pivotal role in optimizing its effectiveness and specificity in clinical applications. The result shows a comparison between the physicochemical properties of the selected phytocompound and standard medications, indicating whether they pass the analysis. Typically, a lead candidate that is taken orally should not exceed 1 violation of Lipinski's rule, as this would negatively impact its bioavailability [20].

### 3.3 ADME (A: absorption, D: distribution, M: metabolism, and E: excretion) Analysis

The pharmacokinetic feasibility of all chosen phytocompounds (as a potential primary candidate) was determined using the SwissADME program available online [21]. The LogP value signifies the lipophilic (lipid-soluble) character of the phytocompound, indicating its ability to be absorbed effectively *via* the skin. Curiously, only three phytocompounds and conventional medicines demonstrated both Blood-Brain Barrier (BBB) permeability and acted as Permeability-glycoprotein (P-gp) substrates, unlike the other phytocompounds. P-gp, an ATP-dependent efflux transporter, eliminates pharmaceuticals from biological systems. The normal elimination of pharmaceutical medications from the body through the gut by P-gp decreases the effectiveness of these drugs in terms of their pharmacokinetics, especially if they are known to be substrates of P-gp. CYPs (Cytochrome P450 enzymes) are a group of metabolic enzymes that are involved in the transformation of xenobiotics. Compounds that hindered the activity of CYPs would result in higher levels of that compound in the bloodstream, therefore leading to increased bioavailability. Kp, also known as skin permeability, is mostly utilized to quantify the process of chemical penetration through the epidermis, which is the outermost layer of the skin [22].

## 4 DISCUSSION

Cervical cancer is one of the four most common cancers among women and is characterized by high incidence and mortality rates of 21.1% and 22.1%, respectively. Cervical cancer is the second most frequent malignancy and the second largest cause of cancer-related deaths among women in less developed nations, behind breast cancer [ 23 ]. The Human Papillomavirus (HPV) is the main cause of cervical cancer, accounting for 79% to 100% of cases. About 70% of these are associated with HPV-16 and HPV-18, two high-risk HPV subtypes [ 24 ]. Traditional chemotherapy often faces challenges such as drug resistance, which can reduce its long-term effectiveness. This has fueled growing interest in natural compounds as potential cancer treatments. Cervical and other malignancies are being aggressively treated with inexpensive natural substances that have a strong anticancer potential and minimal toxicity. Flavonoids have emerged as a primary focus of research, showing significant reductions in cell viability *in vitro.* This effect is attributed, in part, to the inhibition of the oncoprotein E6, which leads to increased p53 levels and subsequent induction of apoptosis [ 25 ]. Flavonoids are a type of phenolic compound present in fruits, vegetables, and plants. Their C6-C3-C6 carbon skeleton structure sets them apart. These substances have attracted considerable scientific attention and are frequently found in the human diet. According to reports, they have anti-inflammatory, anti-microbial, antiviral, antioxidant, antidiabetic, and anti-cancer properties [ 26 ].

Several studies show that plant-derived compounds such as rutin, hesperidin, apigenin, genistein, naringenin, and naringin have demonstrated therapeutic effects against several cancer types. These benefits highlight the urgent need to identify biologically active, naturally-derived compounds for the treatment of cervical cancer. The potential of natural plant products as chemotherapeutic drugs that block numerous cancer-causing pathways has garnered significant attention.

Recent research has highlighted the significance of the Notch signaling pathway in various carcinomas. Notch signaling regulates cell division, differentiation, and survival, among other functions in various cell types and environments [27]. Notch pathway dysregulation has been linked to cervical cancer and has been demonstrated to be crucial to the development of malignancy. Several studies report increased Notch expression in cervical carcinoma cells. However, recent findings suggest that Notch1 is downregulated in late-stage HPV-infected tumors and that Notch signaling may counteract HPV-induced transformation. This highlights the complex relationship between Notch signaling and cervical carcinogenesis [28].

Consequently, our focus lies in screening natural compounds (flavonoids) that specifically target the Notch signaling pathway in cervical cancer. However, additional investigation regarding their mode of action and *in silico* efficacy is necessary to develop broad-spectrum anticancerous agents of clinical relevance [29]. One of the greatest techniques for computer-based drug design is molecular docking, through which novel ligands for receptors of known structure can be designed, and their interaction energies can be calculated. Recently, there has been a notable increase in the number of reports documenting the successful utilization of *in silico* studies within drug development programs across various therapeutic domains [30].

The current research used the crystal structures of the targeted proteins, jagged 2, notch 2, and notch 3, for molecular docking with phytocompounds. To identify effective phytoinhibitors for the notch signaling pathway, a total of 25 phytocompounds with anticancerous activity were selected from the PubChem database and docked with the jagged 2, notch 2, and notch 3 protein structures using AutoDockVina tools. Docking results were analyzed by binding energy and hydrogen bond formation to identify potent phytocompounds as the optimal ligands. Among the top 25 flavanones, the five most prominent are hesperidin, naringenin, naringin, didymin, and eriocitrin. However, only naringenin complies with Lipinski's rule of five, making it the preferred choice for further research. The binding energy of targeted proteins (jagged 2, notch 2, and notch 3) with naringenin was -6.3,-5.5, and -8.5, respectively. The interactions observed between the tested flavonoids and the target protein align with findings from similar *in silico* studies, where flavonoids establish a range of non-covalent interactions within the binding pocket. Typically, hydrogen bonding plays a crucial role, with hydroxyl and carbonyl groups of flavonoids acting as hydrogen bond donors and acceptors, respectively. Additionally, hydrophobic interactions are commonly observed between the aromatic rings of flavonoids and nonpolar residues, stabilizing the ligand within the active site. Electrostatic interactions, particularly with charged amino acid residues, contribute further to Interaction strength. The number of interactions varies depending on the flavonoid structure, with polyhydroxylated flavonoids generally forming more hydrogen bonds. These findings are consistent with previous molecular docking studies, which highlight the structural diversity of flavonoids as a key factor influencing their binding mode and interaction profile [31]. Numerous studies indicate that naringenin plays a multifaceted role in its potential to combat cancer [32]. It suppresses cancer cell invasion, metastasis, angiogenesis, proliferation, and migration while also promoting apoptosis and autophagy. Its ability to modulate various molecular signaling pathways and targets involved in carcinogenesis underlies its effectiveness against cervical cancer. Naringenin has several benefits, including minimal toxicity, wide availability, and low cost, making it suitable for treating cervical carcinomas [33]. This method reduces the time and costs of designing a drug as well as analyzing the drug's likeliness. Consequently, our study corroborated the efficacy of these compounds against cervical cancer, suggesting their potential as viable therapeutic agents [34]. More *in vitro* studies are needed to validate its candidature for cervical cancer management. Clinical trials are also needed to further confirm its significance in preparing a lead candidate for cervical cancer.

## CONCLUSION

The focus of this study was to use computer-based methods to uncover powerful plant-based compounds that can block the Notch signaling system. This study aimed to investigate their potential as cancer treatments. Flavonoids offer a varied approach for treating cervical carcinogenesis by specifically targeting important parts of the Notch signaling system. This technique has the potential to provide advantages in terms of its effectiveness, safety, and tolerability. Computational investigations demonstrated that naringenin exhibited superior binding energy and robust inhibitory constant compared to the standard drug examined in this study. Further *in vitro* investigations are necessary to corroborate these findings and confirm the physiological significance of these findings.

## Figures and Tables

**Table 1:** List of ligands.

S. No	Ligands	PUBCHEM ID	Structure
1.	BLUMEATIN Source:- *Blumea balsamifera* (Ngai camphor and sambong)	70696494	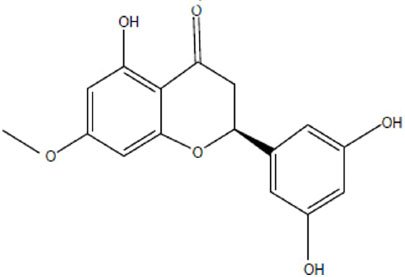
2.	BRUTIERIDIN Source:- *Citrus bergamia* (bergamot orange)	273872997	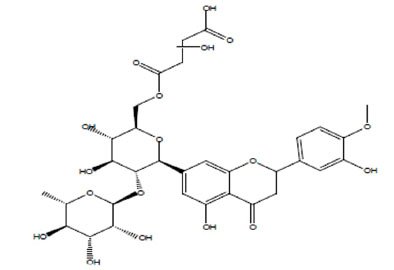
3.	BUTIN Source:- seeds of *Vernonia anthelmintica* (Asteraceae) and in the wood of *Dalbergia odorifera* (Fabaceae)	92775	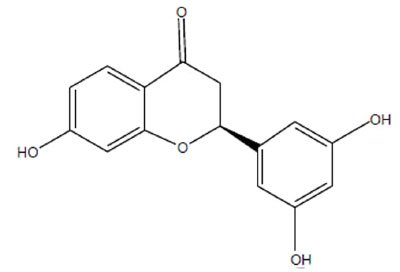
4.	DIDYMIN Source:- extract of the Acacia tree	16760075	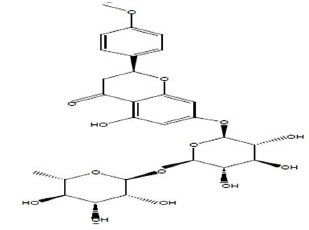
5.	ERIOCITRIN Source:- *Citrus limon*	83489	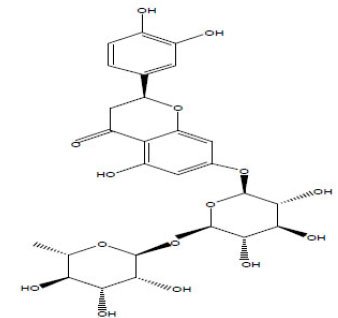
6.	ERIODICTYOL Source:- *Eupatorium album, Eupatorium hyssopifolium*	440735	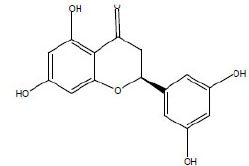
7.	HESPERETIN Source:- *Dalbergia parviflora.*	72281	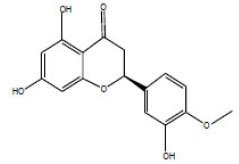
8.	HESPERIDIN Source:- *Citrus tankan*	10621	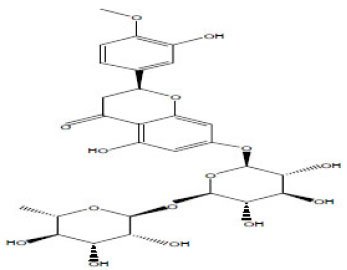
9.	HOMOERIODICTYOL Source:- *Smilax corbularia, Limonium aureum*	73635	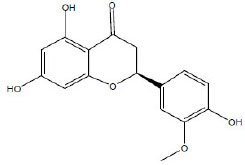
10.	ISOSAKURANETIN Source:- *Ageratina altissima, Chromolaena odorata*.	160481	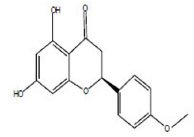
11.	MELITIDIN Source:- *Citrus grandis ‘Tomentosa’*.	101485562	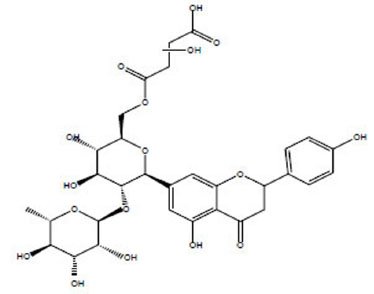
12.	NARINGENIN Source:- *Elaeodendron croceum, Garcinia multiflora*.	439246	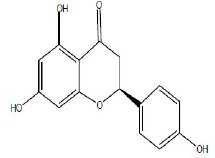
13.	NARINGIN Source:- *Podocarpus fasciculus, Citrus latipes.*	442428	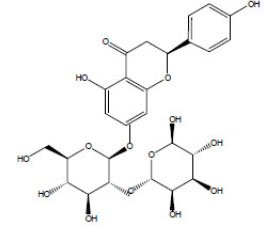
14.	NARIRUTIN Source:- *Cyclopia subternata, Citrus latipes.*	442431	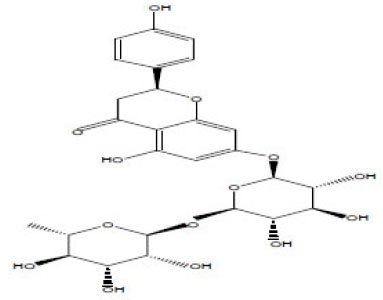
15.	NEOERIOCITRIN Source:- *Citrus latipes, Citrus hystrix.*	114627	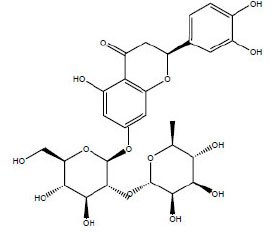
16.	NEOHESPERIDIN Source:- *Citrus medica, Arabidopsis thaliana.*	442439	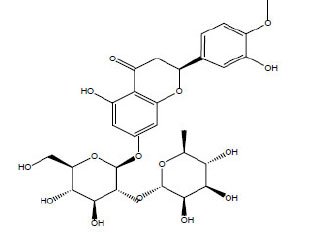
17.	LIQUIRITIGENIN Source:- *Dracaena draco, Pterocarpus marsupium.*	114829	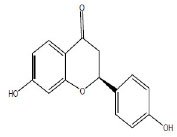
18.	PINOCEMBRIN Source:- *Prunus leveilleana, Alpinia rafflesiana.*	68071	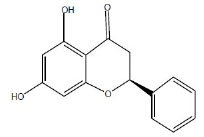
19.	PONCIRIN Source:- *Citrus medica, Micromeria graeca.*	442456	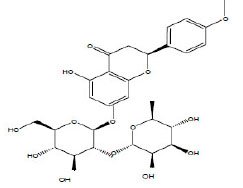
20.	PINOSTROBIN Source:- *Taxandria spathulata, Onychium siliculosum.*	73201	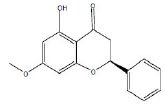
21.	PRUNIN Source:- *Prunus mume, Podocarpus nivalis.*	92794	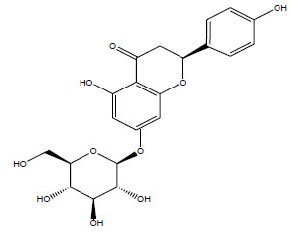
22.	RUTIN Source:-	78901	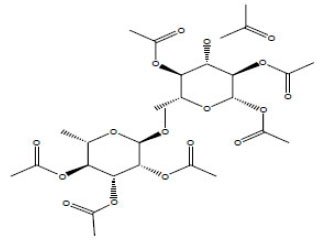
23.	SAKURANETIN Source:- *Ageratina altissima, Chromolaena odorata.*	73571	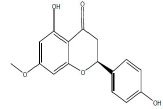
24.	SAKURANIN Source:- *Prunus cerasus, Pyracantha coccinea.*	73607	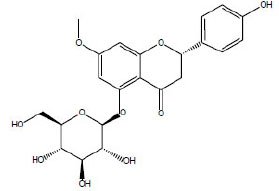
25.	STERUBIN Source:- *Artemisia halodendron, Traversia baccharoides.*	1268276	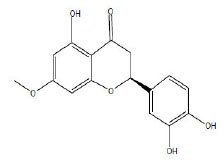

**Table 2:** Results of the blind docking with jagged 2.

S. No.	FLAVANONES	Vina Score (kcal/mol)	Cavity Size	X-axis	Y-axis	Z-axis	Structure
1.	BLUMEATIN	-6.3	181	39	89	32	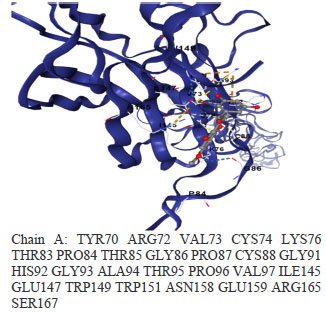
2.	BRUTIERIDIN	-7.3	560	61	88	30	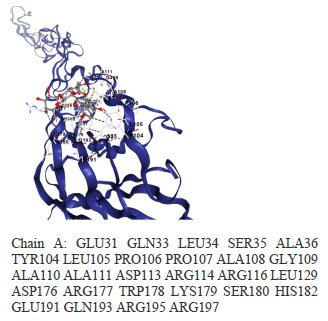
3.	BUTIN	-6.2	560	61	88	30	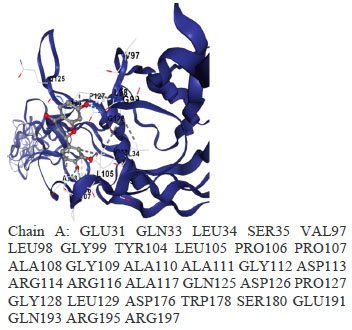
4.	DIDYMIN	-7.4	560	61	88	30	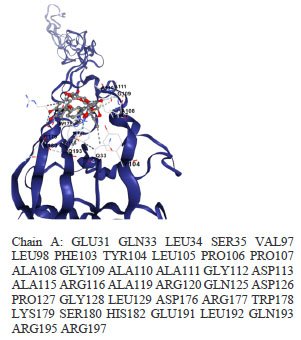
5.	ERIOCITRIN	-7.4	560	61	88	30	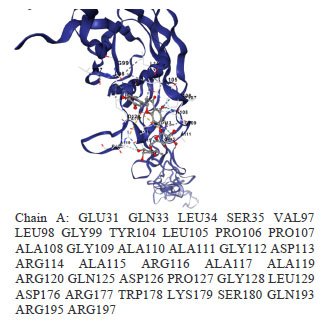
6.	ERIODICTYOL	-6.2	560	61	88	30	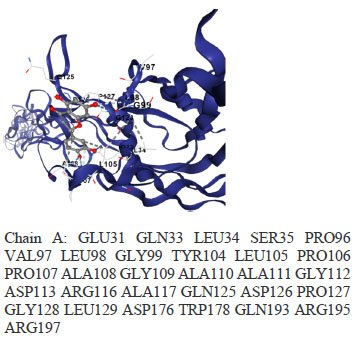
7.	HESPERETIN	-6.2	560	61	88	30	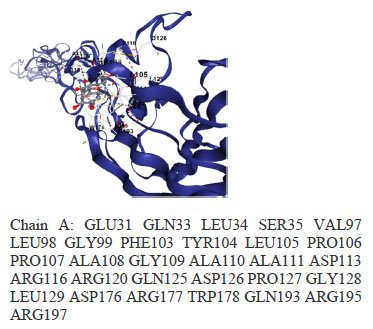
8.	HESPERIDIN	-7.8	200	67	81	-17	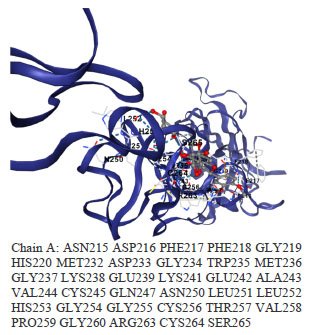
9.	HOMOERIODICTYOL	-6.2	560	61	88	30	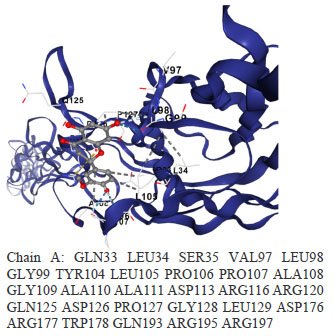
10.	ISOSAKURANETIN	-6.1	560	61	88	30	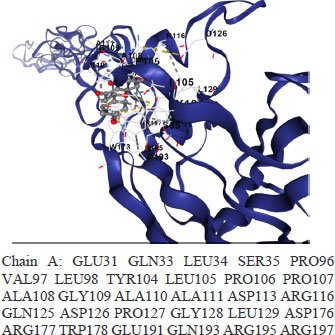
11.	MELITIDIN	-6.9	200	67	81	-17	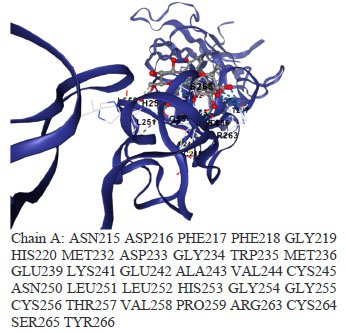
12.	NARINGENIN	-6.3	560	61	88	30	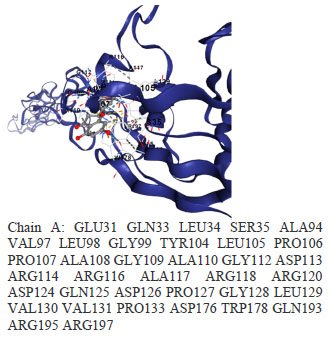
13.	NARINGIN	-7.2	200	67	81	-17	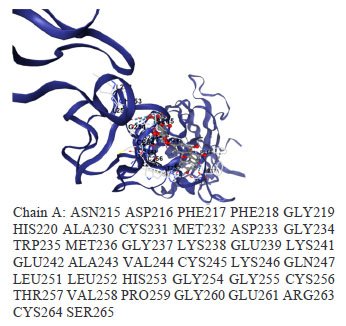
14.	NARIRUTIN	-7.6	560	61	88	30	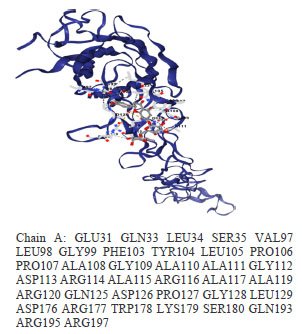
15.	NEOERIOCITRIN	-7.6	173	42	84	52	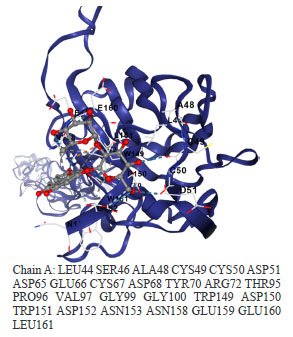
16.	NEOHESPERIDIN	-6.9	560	61	88	30	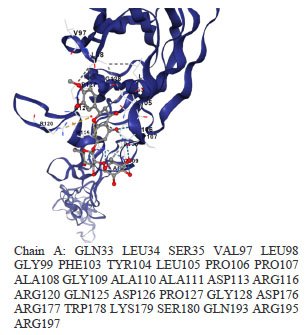
17.	LIQUIRITIGENIN	-6.2	560	61	88	30	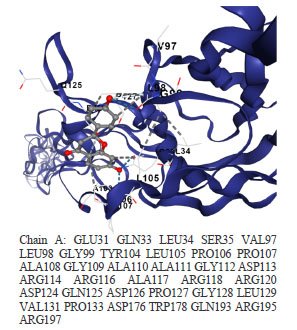
18.	PINOCEMBRIN	-6.2	560	61	88	30	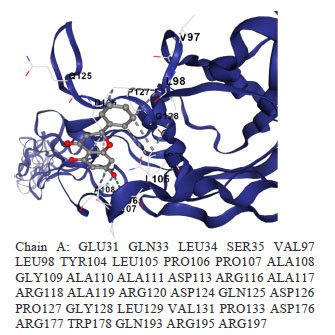
19.	PONCIRIN	-7.1	560	61	88	30	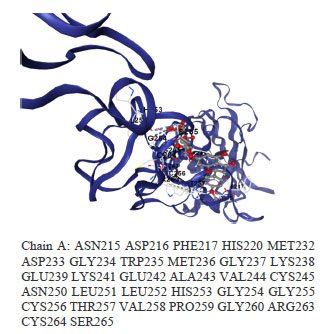
20.	PINOSTROBIN	-6.1	560	61	88	30	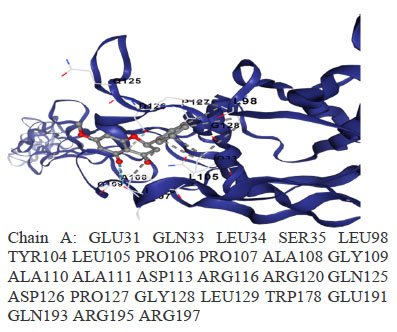
21.	PRUNIN	-6.0	181	39	89	32	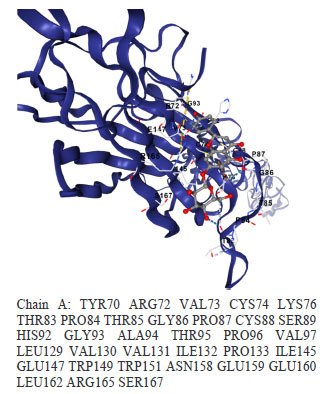
22.	RUTIN	-5.7	200	67	81	-17	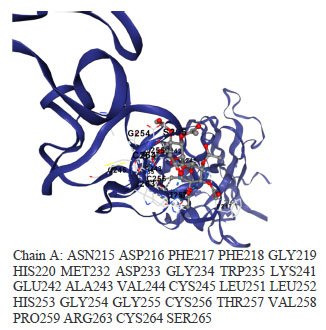
23.	SAKURANETIN	-6.2	560	61	88	30	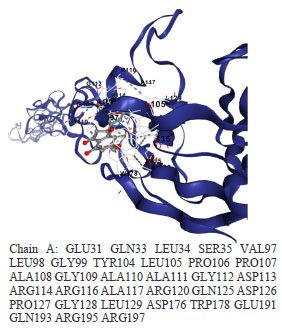
24.	SAKURANIN	-6.3	200	67	81	-17	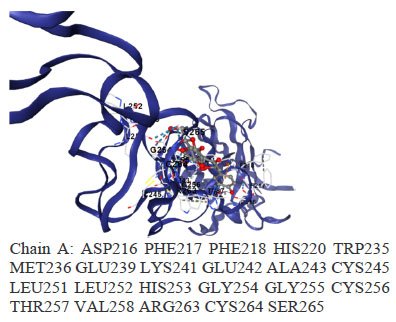
25.	STERUBIN	-6.2	560	61	88	30	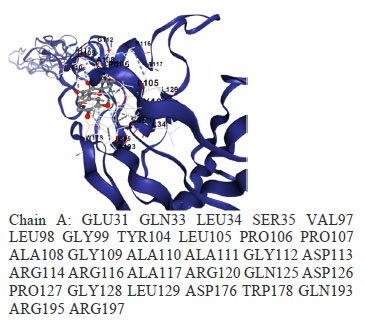

**Table 3:** Results of the blind docking with notch 2.

S. No.	FLAVANONES	Vina Score (kcal/mol)	Cavity Size	X-axis	Y-axis	Z-axis	Structure
1.	BLUMEATIN	-5.2	28	-33	-18	-44	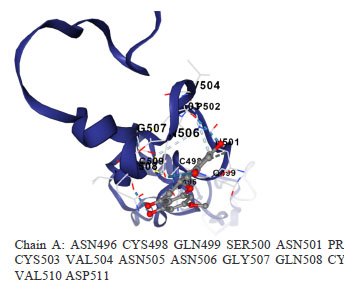
2.	BRUTIERIDIN	-7.0	13	-11	-15	16	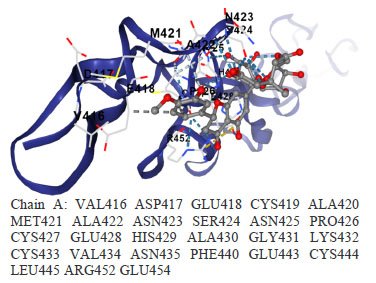
3.	BUTIN	-5.5	13	-11	-15	16	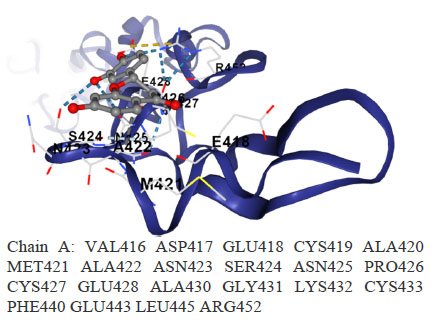
4.	DIDYMIN	-6.9	13	-11	-15	16	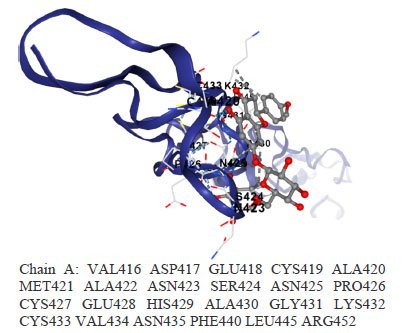
5.	ERIOCITRIN	-6.9	13	-11	-15	16	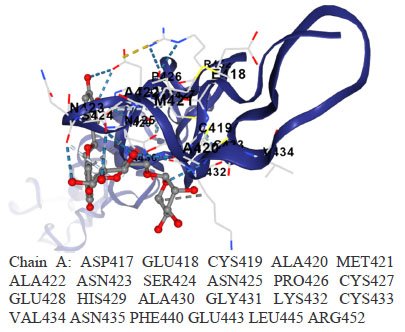
6.	ERIODICTYOL	-5.2	13	-11	-15	16	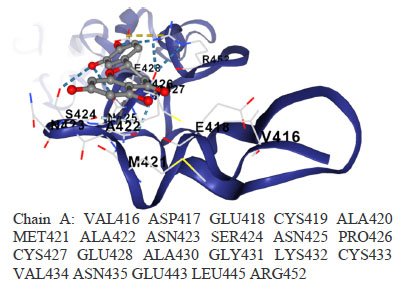
7.	HESPERETIN	-5.5	28	-33	-18	-44	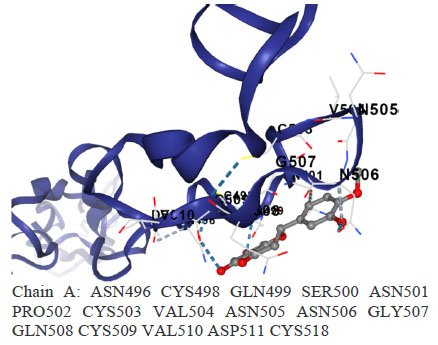
8.	HESPERIDIN	-6.9	26	-3	-22	9	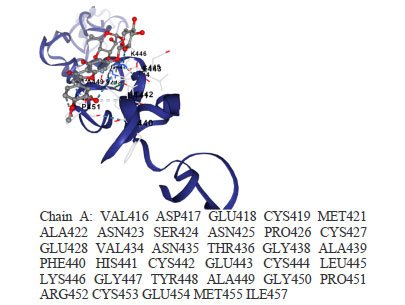
9.	HOMOERIODICTYOL	-5.5	13	-11	-15	16	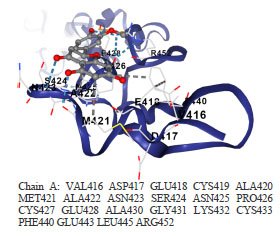
10.	ISOSAKURANETIN	-5.5	28	-33	-18	-44	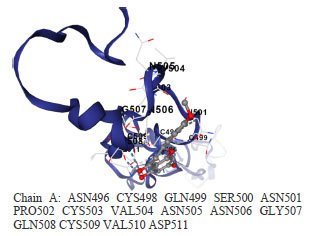
11.	MELITIDIN	-6.7	13	-11	-15	16	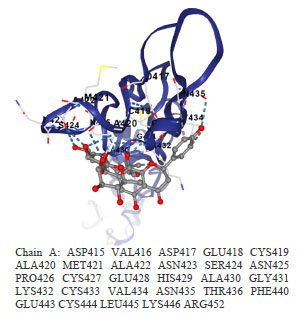
12.	NARINGENIN	-5.5	13	-11	-15	16	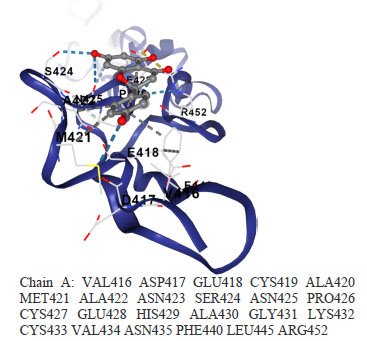
13.	NARINGIN	-6.6	28	-33	-18	-44	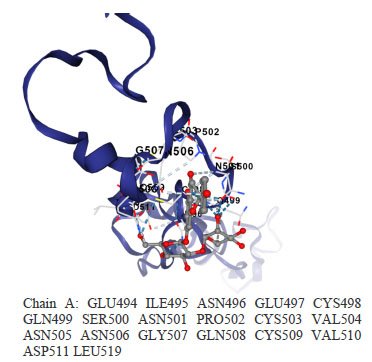
14.	NARIRUTIN	-6.6	28	-33	-18	-44	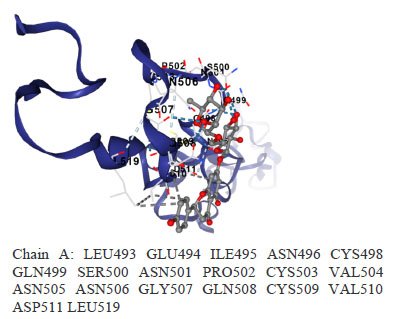
15.	NEOERIOCITRIN	-6.6	24	-14	-16	-32	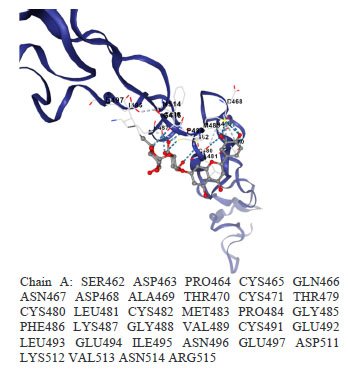
16.	NEOHESPERIDIN	-6.4	28	-33	-18	-44	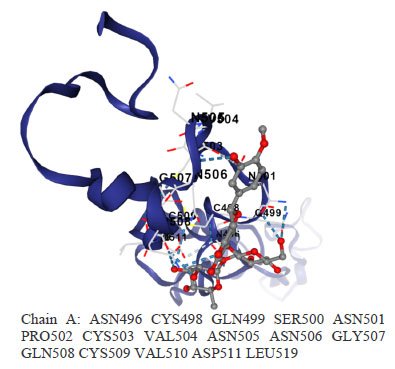
17.	LIQUIRITIGENIN	-5.2	13	-11	-15	16	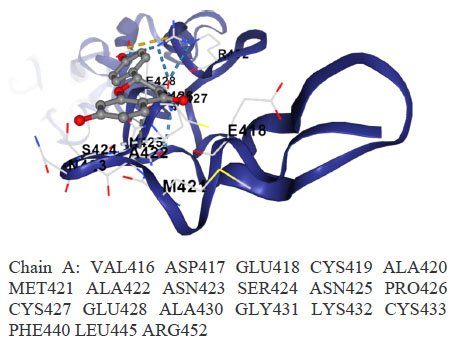
18.	PINOCEMBRIN	-5.5	13	-11	-15	16	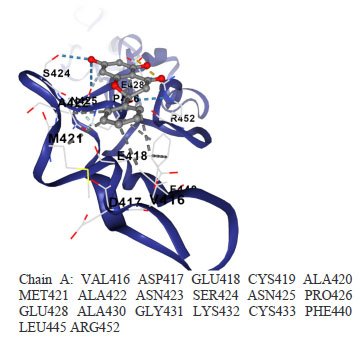
19.	PONCIRIN	-6.9	26	-3	-22	9	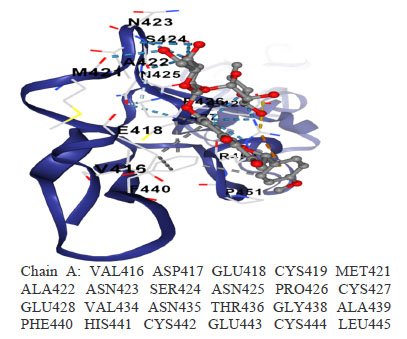
20.	PINOSTROBIN	-5.4	13	-11	-15	16	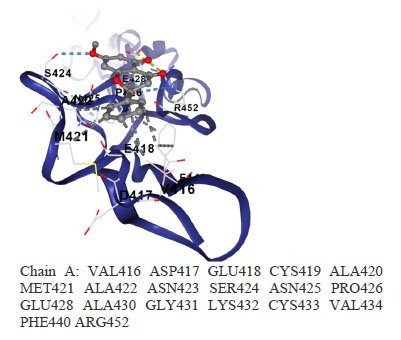
21.	PRUNIN	-5.2	13	-11	-15	16	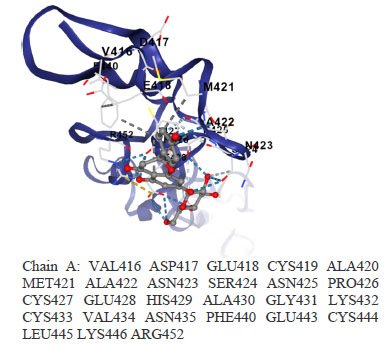
22.	RUTIN	-4.8	28	-33	-18	-44	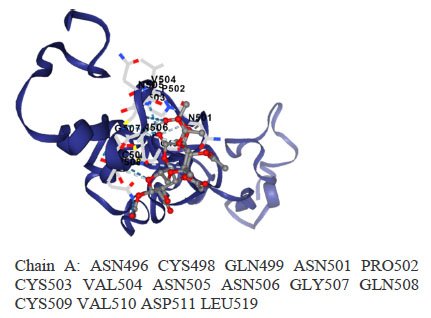
23.	SAKURANETIN	-5.2	13	-11	-15	16	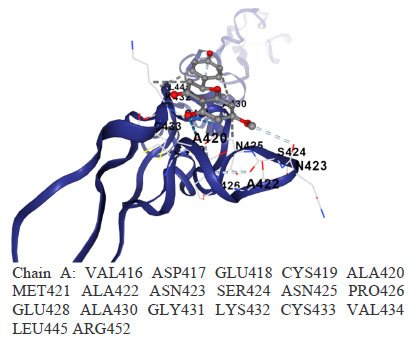
24.	SAKURANIN	-5.3	28	-33	-18	-44	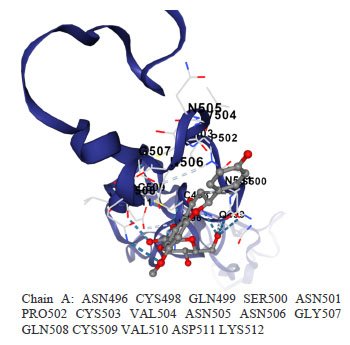
25.	STERUBIN	-5.5	13	-11	-15	16	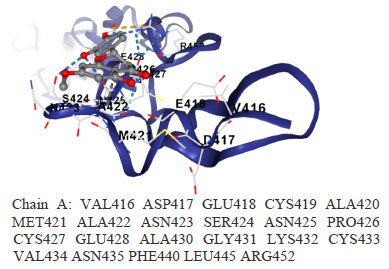

**Table 4:** Results of the blind docking with notch 3.

S. No.	FLAVANONES	Vina Score (kcal/mol)	Cavity Size	X-axis	Y-axis	Z-axis	Structure
1.	BLUMEATIN	-8.2	458	108	5	-2	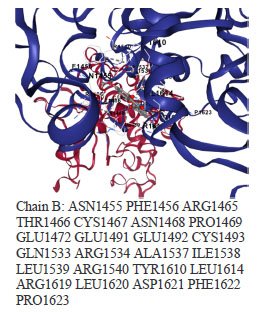
2.	BRUTIERIDIN	-8.5	458	108	5	-2	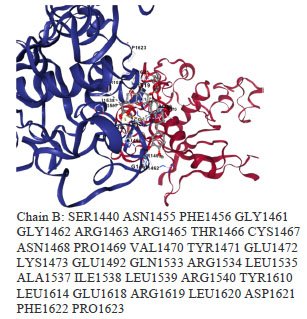
3.	BUTIN	-8.3	378	109	-22	-28	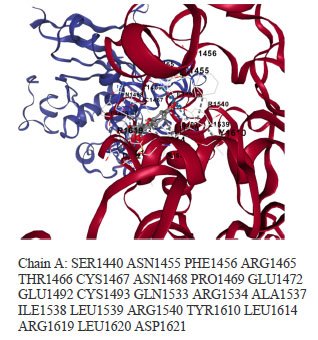
4.	DIDYMIN	-9.4	378	109	22	-28	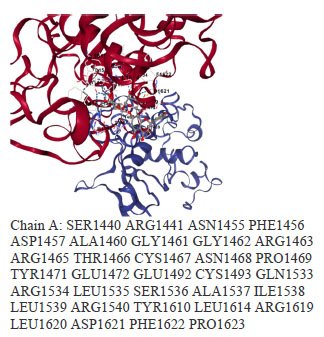
5.	ERIOCITRIN	-9.3	458	108	5	-2	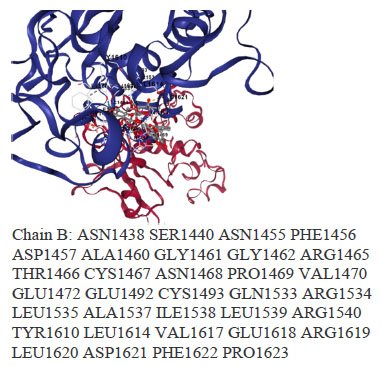
6.	ERIODICTYOL	-8.4	378	109	22	-28	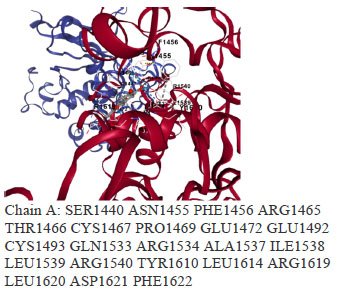
7.	HESPERETIN	-8.4	458	108	5	-2	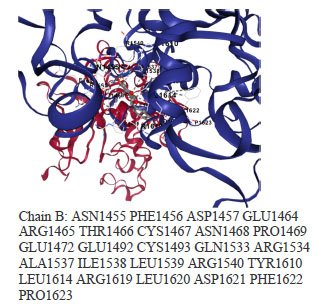
8.	HESPERIDIN	-9.8	378	109	22	-28	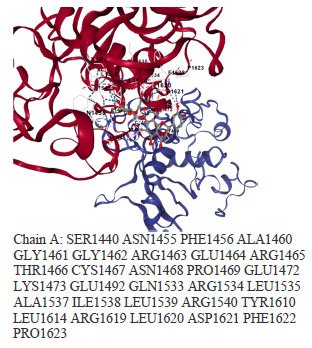
9.	HOMOERIODICTYOL	-8.2	458	108	5	-2	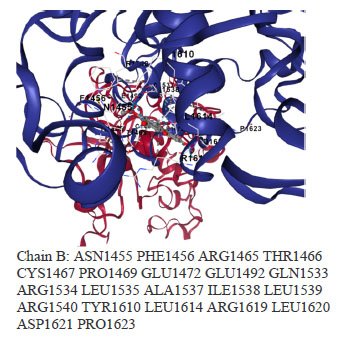
10.	ISOSAKURANETIN	-8.3	458	108	5	-2	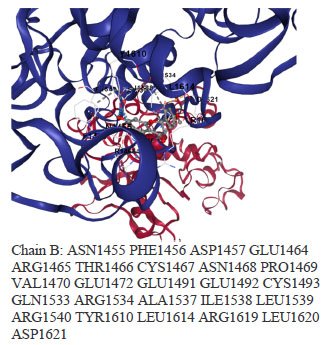
11.	MELITIDIN	-8.5	458	108	5	-2	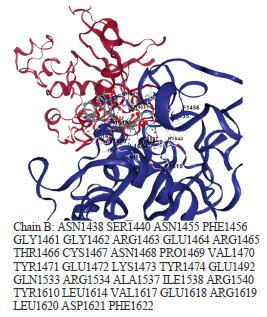
12.	NARINGENIN	-8.5	378	109	22	-28	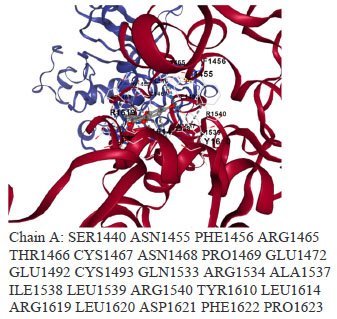
13.	NARINGIN	-8.8	378	109	22	-28	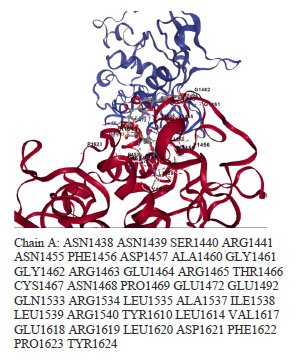
14.	NARIRUTIN	-9.3	458	108	5	-2	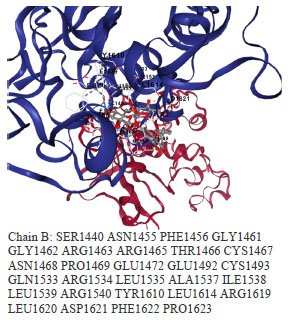
15.	NEOERIOCITRIN	-9.4	378	109	22	-28	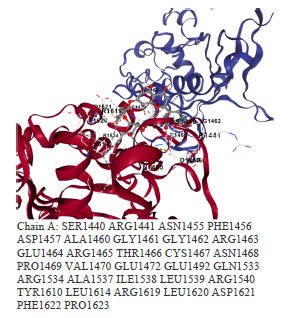
16.	NEOHESPERIDIN	-10	608	97	5	9	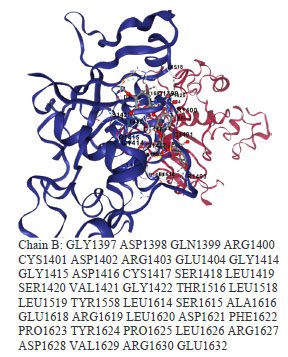
17.	LIQUIRITIGENIN	-8.2	378	109	22	-28	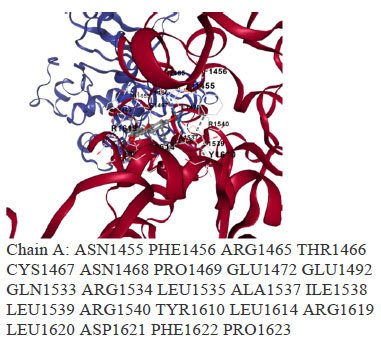
18.	PINOCEMBRIN	-8.2	378	109	22	-28	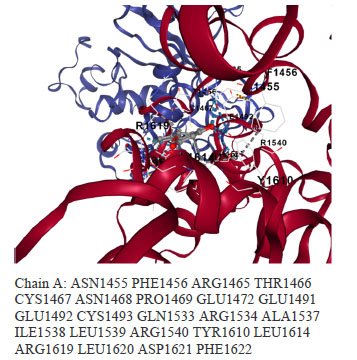
19.	PONCIRIN	-9.5	378	109	22	-28	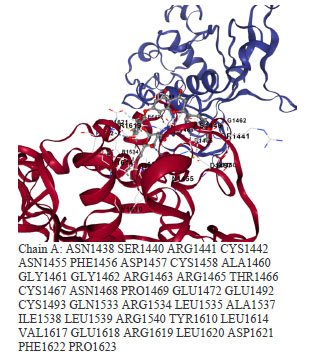
20.	PINOSTROBIN	-8.1	458	108	5	-2	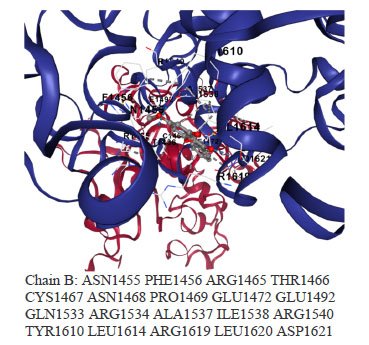
21.	PRUNIN	-8.3	458	108	5	-2	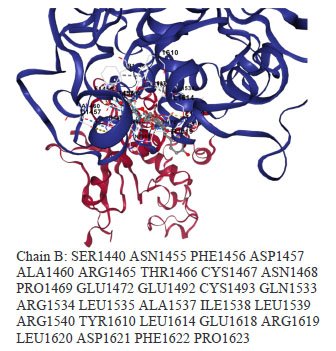
22.	RUTIN	-7.1	378	109	22	-28	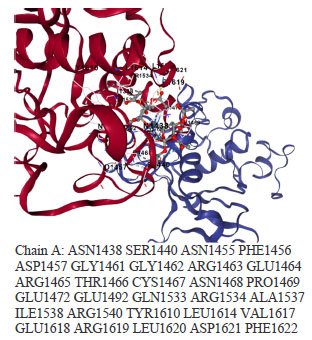
23.	SAKURANETIN	-8.2	458	108	5	-2	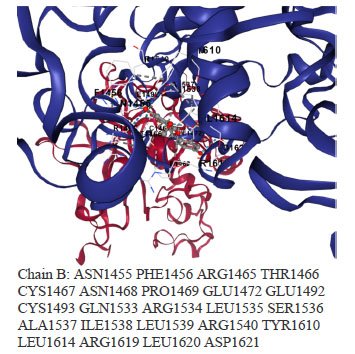
24.	SAKURANIN	-8.3	378	109	22	-28	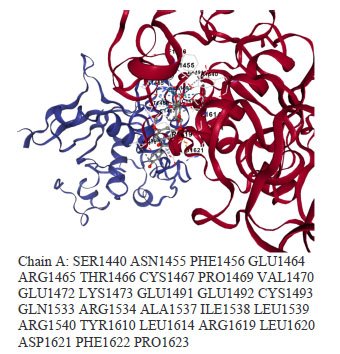
25.	STERUBIN	-8.2	458	108	5	-2	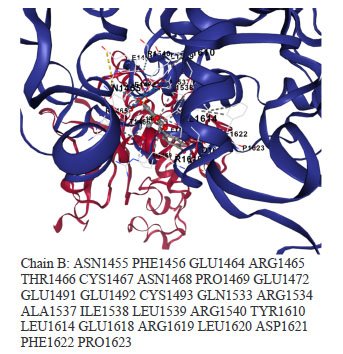

**Table 5:** Docking with cisplatin PubChem CID 5702198 (standard drug) for cervical cancer.

S. No	CISPLATIN	Vina Score (kcal/mol)	Cavity Size	X-axis	Y-axis	Z-axis	Structure
1.	JAGGED 2	-1.0	543	-25	8	-12	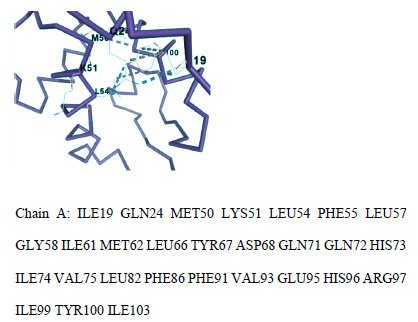
2.	NOTCH 2	-1.1	26	-3	-22	9	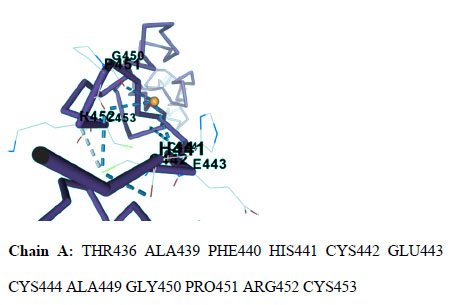
3.	NOTCH 3	-1.2	458	108	5	-2	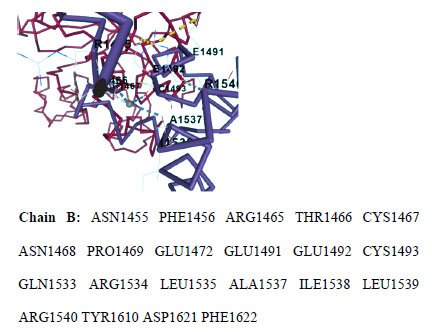

**Table 6:** Drug likeness.

S. No.	Flavanones	Lipinski	Ghose	Veber	Egan	Muegge	Bioavailability Score
1.	Blumeatin	Yes	Yes	Yes	Yes	Yes	0.55
2.	Brutieridin	No	No	No	No	No	0.11
3.	Butin	Yes	Yes	Yes	Yes	Yes	0.55
4.	Didymin	No	No	No	No	No	0.17
5.	Eriocitrin	No	No	No	No	No	0.17
6.	Eriodictyol	Yes	Yes	Yes	Yes	Yes	0.55
7.	Hesperetin	Yes	Yes	Yes	Yes	Yes	0.55
8.	Hesperidin	No	No	No	No	No	0.17
9.	Homoeriodictyol	Yes	Yes	Yes	Yes	Yes	0.55
10.	Isosakuranetin	Yes	Yes	Yes	Yes	Yes	0.55
11.	Melitidin	No	No	No	No	No	0.11
12.	Naringenin	Yes	Yes	Yes	Yes	Yes	0.55
13.	Naringin	No	No	No	No	No	0.17
14.	Narirutin	No	No	No	No	No	0.17
15.	Neoeriocitrin	No	No	No	No	No	0.17
16.	Neohesperidin	No	No	No	No	No	0.17
17.	Liquiritigenin	Yes	Yes	Yes	Yes	Yes	0.55
18.	Pinocembrin	Yes	Yes	Yes	Yes	Yes	0.55
19.	Poncirin	No	No	No	No	No	0.17
20.	Pinostrobin	Yes	Yes	Yes	Yes	Yes	0.55
21.	Prunin	Yes	Yes	No	No	No	0.55
22.	Rutin	No	No	No	No	No	0.17
23.	Sakuranetin	Yes	Yes	Yes	Yes	Yes	0.55
24.	Sakuranin	Yes	Yes	No	No	No	0.55
25.	Sterubin	Yes	Yes	Yes	Yes	Yes	0.55

**Table 7:** ADME analysis.

S. No.	Flavanones	PubChem Id	GI Absorption	BBB Permeant	P-gp Substrate	CYP2C19 Inhibitor	Log Kp (Skin Permeation)
1.	Blumeatin	70696494	High	No	Yes	No	-6.31 cm/s
2.	Brutieridin	273872997	Low	No	Yes	No	-12.10 cm/s
3.	Butin	92775	High	No	Yes	No	-6.58 cm/s
4.	Didymin	16760075	Low	No	Yes	No	-10.40 cm/s
5.	Eriocitrin	83489	Low	No	Yes	No	-10.90 cm/s
6.	Eriodictyol	440735	High	No	Yes	No	-6.62 cm/s
7.	Hesperetin	72281	High	No	Yes	No	-6.30 cm/s
8.	Hesperidin	10621	Low	No	Yes	No	-10.12 cm/s
9.	Homoeriodictyol	73635	High	No	Yes	No	-6.30 cm/s
10.	Isosakuranetin	160481	High	Yes	No	Yes	-6.02 cm/s
11.	Melitidin	101485562	Low	No	Yes	No	-11.89 cm/s
12.	Naringenin	439246	High	No	Yes	No	-6.17 cm/s
13.	Naringin	442428	Low	No	Yes	No	-10.15 cm/s
14.	Narirutin	442431	Low	No	Yes	No	-10.54 cm/s
15.	Neoeriocitrin	114627	Low	No	Yes	No	-10.51 cm/s
16.	Neohesperidin	442439	Low	No	Yes	No	-10.36 cm/s
17.	Liquiritigenin	114829	High	Yes	Yes	No	-6.23 cm/s
18.	Pinocembrin	68071	High	Yes	No	Yes	-5.82 cm/s
19.	Poncirin	442456	Low	No	Yes	No	-10.00 cm/s
20.	Pinostrobin	73201	High	Yes	No	Yes	-5.68 cm/s
21.	Prunin	92794	Low	No	Yes	No	-8.49 cm/s
22.	Rutin	78901	Low	No	No	No	-10.24 cm/s
23.	Sakuranetin	73571	High	Yes	No	Yes	-6.02 cm/s
24.	Sakuranin	73607	Low	No	Yes	No	-8.73 cm/s
25.	Sterubin	1268276	High	No	Yes	No	-6.48 cm/s

## Data Availability

All data generated or analyzed during this study are included in this published article.

## LIST OF ABBREVIATIONS

HPV: Human Papillomavirus
PDB: Protein Data Bank
MW: Molecular Weight
ADME: Absorption, Distribution, Metabolism, and Excretion
TPSA: Topological Polar Surface Area
BBB: Blood-Brain Barrier
